# Hydrologic regime alteration and influence factors in the Jialing River of the Yangtze River, China

**DOI:** 10.1038/s41598-022-15127-4

**Published:** 2022-07-01

**Authors:** Wenxian Guo, Xuyang Jiao, Haotong Zhou, Yongwei Zhu, Hongxiang Wang

**Affiliations:** 1 College of Water Resources, North China University of Water Resources and Electric Power, Zhengzhou, 450045 China; 2 School of Water Conservancy, North China University of Water Resources and Electric Power, Zhengzhou, 450045 China

**Keywords:** Ecology, Environmental sciences, Environmental social sciences, Hydrology

## Abstract

Under the influence of climate alteration and human activities, the hydrological regime of rivers is changing dramatically, which has become a hot spot for water environment research. In this study, we quantitatively assessed the degree of hydrological variability of the Jialing River and the contribution of climate alteration and human activities to hydrological alterations using the ecohydrological indicator range of alteration (IHA-RVA) method and Budyko hypothesis formulations. The results showed that (1) The average annual runoff and the precipitation of Jialing River showed a decreasing trend, the potential evapotranspiration showed an increasing trend. (2) Compared with before the hydrological situation changed, the degree of alteration in the annual extreme streamflow is 31%, which is a low degree of alteration; the degree of alteration in monthly streamflow, annual extreme flow magnitude, extreme flow ephemeris, and streamflow alteration frequency are 51%, 43%, 54%, and 64% respectively, which are all moderate degrees of alteration; the overall hydrological alteration is 50%, which belongs to moderate alteration. (3) The contribution of precipitation, potential evapotranspiration, and human activities to the runoff alteration is 61%, − 16%, and 55%, respectively. This study provides corresponding references for ecological restoration and sustainable development of the Yangtze River Basin in China.

## Introduction

The river hydrology regime is a dynamic feature of its ecosystem that determines river materials and energy exchange. Alterations in the hydrological regime of rivers will directly affect the stability of ecosystems in the basin and the utilization and development of regional water resources^1^. With increasing climate change and human disturbances, the hydrological regimes of globally important rivers, such as the Amazon^2^, Mississippi^3^, Nile^4^, Yellow River^5^, and Yangtze^6^, have been altered to some extent. In China, the large-scale construction of reservoirs in the Yangtze River basin since the 1960s, along with the soil and water conservation work in the Yangtze River basin, these large-scale human activities are bound to seriously disturb the alterations in the hydrological regime of the Yangtze River^7^. As one of the main tributaries of the upper reaches of the Yangtze River, the Jialing River has a significant runoff and precipitation, making it a tributary with a considerable potential for water resources exploitation on the Yangtze River^8^. Therefore, it is of great significance for the ecological restoration and sustainable development of the Yangtze River Basin to study the hydrological alterations of the Jialing River Basin and to evaluate the influence of various influencing factors on runoff qualitative and quantitatively.

Currently, Richter et al. have summarized 33 ecologically significant IHA (Indicators of Hydrologic Alteration) in terms of streamflow, frequency, duration, timing, and rate of alteration to summarize the overall characteristics of river hydrologic phenomenon^9^. On this basis, the Range of Variability Approach (RVA) was proposed to quantitatively evaluate the degree of hydrologic regime alteration of one or more elements under specific activities^10, 11^. The results can better reflect the degree of hydrologic alteration. Guo et al.^12^ applied the RVA method to study the hydrological variability and river–lake relationship between Poyang Lake and the middle and lower reaches of the Yangtze River. Guo et al.^13^ assessed the analysis of the extent of hydrological alterations in the lower Three Gorges and the impact of hydrological alterations on spawning ground habitat conditions of important fish by the RVA method. Zuo et al.^14^ quantified the effect of dams and sluice gates on the river potential of the Sha Ying River using the RVA method as an example. Mwanza et al.^15^ similarly used this method to analyze the extent of alteration of hydrological indicators at different sections of the Manyame basin in Zimbabwe after the construction of dams. Meanwhile, for the attribution analysis of runoff alterations, scholars in recent years have mainly adopted various hydrological models, cumulative slope rate of alteration, elasticity coefficient method, and other methods to analyze the influencing factors of runoff alterations in different rivers. The elasticity coefficient method based on Budyko's theory incorporates evapotranspiration into climate influencing factors. In contrast, this method has the advantages of easy application and high feasibility, and this method is widely used in essential river basins around the world^16–19^. In summary, studies on the quantitative evaluation of alterations in river hydrological conditions are dominated by IHA, RVA, and the elasticity coefficient method. Their results can better reflect the degree of alteration in rivers after being influenced.

Some scholars have conducted studies on the alterations in the hydrological regime of the Jialing River from different perspectives. Li et al.^20^ analyzed the trend and mutation of Jialing River runoff and sediment based on the hydrological data of long time series in the last 60 years and summarized the role of reservoir construction and ecological engineering on runoff alterations. Liu et al.^21^ based on the longitudinal profile of the main terrace reservoirs of the Jialing River, used GIS methods and digital elevation model data to quantitatively analyze the alterations in the water area, runoff, and sediment after dam impoundment and concluded that the completion of the terrace reservoirs has a significant impact on the hydrological situation of the river and almost determines the trend of the hydrological status of the mainstream section. Zhou et al.^22^ used the double cumulative curve method to quantitatively evaluate the effects of dams, vegetation cover, and precipitation on runoff variability. In summary, most researchers^20, 21^ have limited the identification of runoff variability only to its trend and mutation points. However, there is a lack of in-depth research on the variability of each hydrological index of runoff and the degree. Meanwhile, for the attribution analysis of runoff alterations in the Jialing River, some scholars^22^, although quantitatively evaluating the contribution of different factors, lacked to consider the influence of river evapotranspiration and the response between runoff alterations and each element. At the same time, there is a lack of qualitative analysis of the effects of alterations in hydrological regimes on the aquatic organisms in the Jialing River basin due to the lack of information on marine organisms in the bay, so further research is needed for such issues.

In view of the deficiency of the current research on the hydrological regime alteration of Jialing River, this study is based on the daily hydrological data of Beibei Hydrological Station, a representative hydrological station of Jialing River, from 1956 to 2020. Its main research purposes are: (1) To analyze the long-term series alteration characteristics of hydrological and meteorological elements in the Jialing River basin; (2) Analyze the alteration of the hydrological situation in five aspects of monthly flow, frequency, duration, timing, and variable rate of Jialing River before and after mutation, and comprehensively evaluate the alteration degree of the overall hydrological situation; (3) Quantitatively evaluate the contribution rate of climate alteration and human activities to the alteration of hydrological situation. To provide a scientific basis for the restoration of water environment and water ecology and water resources development and utilization in the upper Yangtze River basin.

## Results

### Trend analysis of hydro-meteorological factors

**Figure 1:**
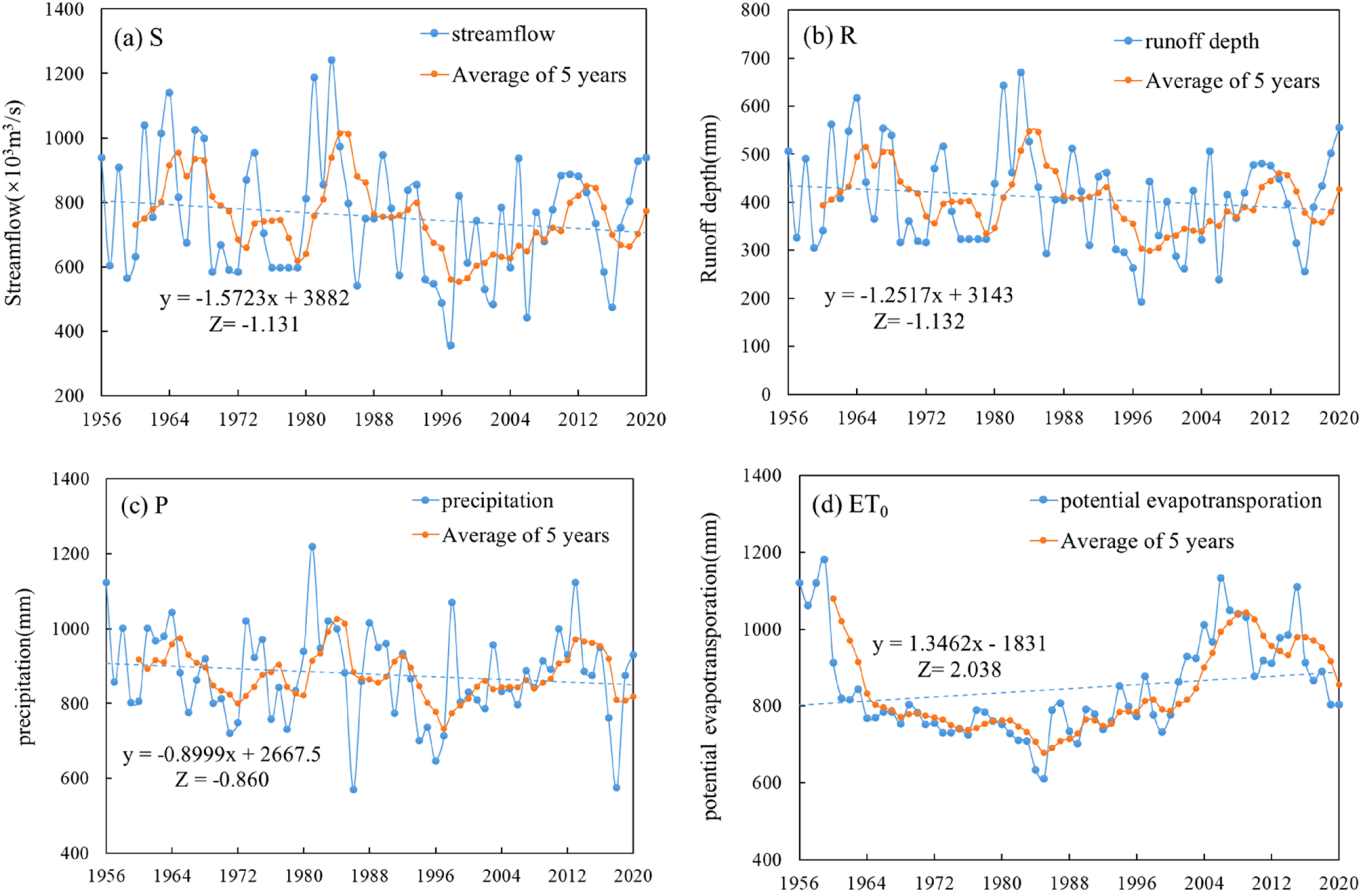
Interannual alteration of (**a**) streamflow, (**b**) runoff depth, (**c**) precipitation and (**d**) potential evapotranspiration.

Figure 1 shows the interannual trends of the average annual streamflow S, the average annual runoff depth R, the average precipitation P and the average potential evapotranspiration ET0 in the Jialing River basin from 1956 to 2020. According to Fig. 1a,b, the annual average streamflow and runoff depth change rate are 1572.3(m^3^/s)/a, 1.251 mm/a, respectively, and the M–K trend test value is around − 1.31, which indicates that the runoff of Jialing River shows a decreasing trend, but not significant. From Fig. 1c, it can be seen that the precipitation change rate is 0.899 mm/a and the M–K trend test value is − 0.86, which indicates that the overall precipitation in the Jialing River basin shows an insignificant decreasing trend. According to Fig. 1d, the rate of change of potential evapotranspiration is 1.3462 mm/a, and the M–K trend test values are all at 2.038, indicating that the potential evapotranspiration in the basin shows a significant increasing trend. According to the 5a sliding average process line, the runoff and precipitation maintain a good consistency, and the overall performance is decreasing, then increasing, then alternately increasing, indicating that the precipitation and runoff are positively correlated and have good synchronization; the potential evapotranspiration shows a decreasing trend before 1985, and the decreasing trend is larger than the precipitation and runoff, then it shows an increase and decreases again in 2010, which is the opposite of the precipitation runoff.

### Analysis of runoff mutation

**Figure 2:**
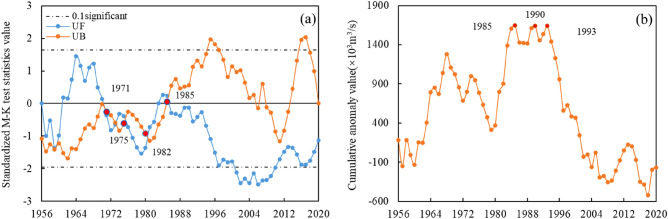
(**a**) M–K mutation test. (**b**) Cumulative anomaly test.

The Mann–Kendall mutation test principle was used to calculate and plot its mutation test curve, while the cumulative distance level method was used for reference, and the results are shown in Fig. 2. According to Fig. 2a, the UF and UB curves intersected several times within the 90% confidence interval, corresponding to 1971, 1975, 1982, and 1985, respectively. The cumulative distance level method was used to synthesize the results to further confirm the mutation years. According to Fig. 2b, the annual mean streamflow showed a significant alteration in the 1980s and turned in 1985, 1990, and 1993, consistent with the M–K test results. The year 1985 was adopted as a mutation in a runoff for careful consideration. The streamflow, runoff depth, precipitation, and potential evaporation alterations were analyzed according to the mutation point. The results are shown in Table 1.

**Table 1:** Mutability analysis of streamflow, runoff depth, precipitation, and potential evaporation.

Period	S (10^3^ m^3^/s)	R (mm)	P (mm)	ET_0_ (mm)
The whole period	756.21	409.22	871.54	845.66
Base period	810.83	431.14	893.71	799.59
Period of alteration	709.39	367.12	842.49	914.44
Amount of alteration	− 101.43	− 64.01	− 51.22	114.84

### Hydrological regime alteration analysis

Referring to the study of Zeng et al. on the environmental changes of Jialing River^23^, and also integrating the analysis results of the mutability test results, the daily runoff periods before 1985 (1956–1985) were taken as the period with weak environmental disturbance, i.e., natural flow series; after 1985 (1986–2020) were taken as the period with strong external disturbance, i.e., flow change series, and on this basis, the IHA-RVA methods were applied to calculate the degree of runoff alteration at Beibei hydrological station before and after the mutation, and the calculation results are shown in Table 2.

**Table 2:** IHA index statistics table before and after a mutation in Jialing River.

IHA indicators		Median value		Hydrological alteration degree	
		Pre-impact	Post-impact	value %	Degree
Group 1	January (m^3^ s^−1^)	433.5	536.5	77.38	H
	February (m^3^ s^−1^)	363.8	415.3	32.14	L
	March (m^3^ s^−1^)	391	532	66.07	H
	April (m^3^ s^−1^)	838.8	823	17.86	L
	May (m^3^ s^−1^)	1668	1695	20.83	L
	June (m^3^ s^−1^)	1565	1375	15.56	L
	July (m^3^ s^−1^)	3650	2605	77.38	H
	August (m^3^ s^−1^)	2975	2270	69.64	H
	September (m^3^ s^−1^)	2733	2435	69.64	H
	October (m^3^ s^−1^)	1990	1745	43.45	M
	November (m^3^ s^−1^)	1085	1128	20.83	L
	December (m^3^ s^−1^)	557	596	20.83	L
Group 2	1-day minimum (m^3^ s^−1^)	328.5	274	66.07	H
	3-day minimum (m^3^ s^−1^)	333.3	296.7	1.786	L
	7-day minimum (m^3^ s^−1^)	336.3	329.1	20.83	L
	30-day minimum (m^3^ s^−1^)	349.2	402.6	57.78	M
	90-day minimum (m^3^ s^−1^)	422.1	515.6	32.14	L
	1-day maximum (m^3^ s^−1^)	22,200	19,950	43.45	M
	3-day maximum (m^3^ s^−1^)	18,680	16,340	20.83	L
	7-day maximum (m^3^ s^−1^)	14,660	12,090	54.76	M
	30-day maximum (m^3^ s^−1^)	7605	5930	26.11	L
	90-day maximum (m^3^ s^−1^)	5178	3815	43.45	M
	Base flow index (m^3^ s^−1^)	0.1541	0.1951	43.45	M
Group 3	Date of minimum (ds)	60	50	31.14	L
	Date of maximum (ds)	200	210	30.67	L
Group 4	Low pulse count (times)	2	13	82.41	H
	Low pulse duration (ds)	37.75	2	63.46	M
	High pulse count (times)	9	9	24.6	L
	High pulse duration (ds)	6	5	16.18	L
Group 5	Rise rate (m^3^ d^−1^)	114.5	108.5	32.14	L
	Fall rate (m^3^ d^−1^)	− 70	− 127	36.67	M
	Number of reversals (times)	102.5	159.5	100	H

H indicates a high degree of alteration, M indicates moderate alteration, L indicates a low degree of alteration.

**Figure 3:**
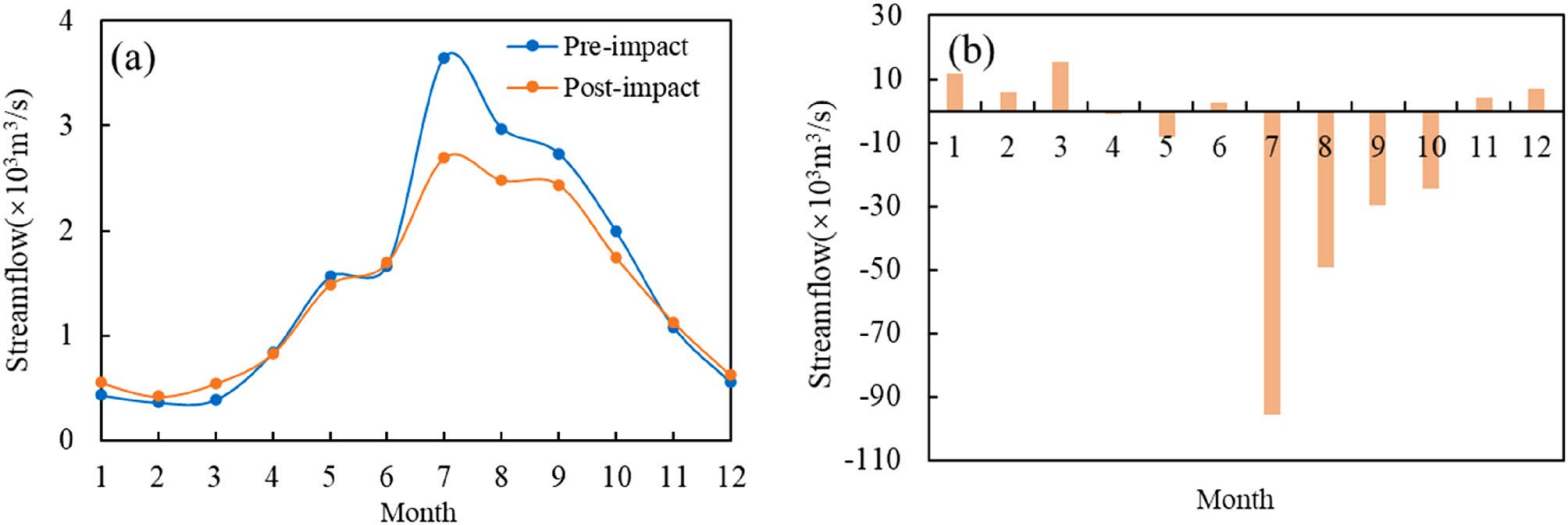
(**a**) Alteration of the monthly streamflow before and after. (**b**) Monthly streamflow difference before and after changing.

**Figure 4:**
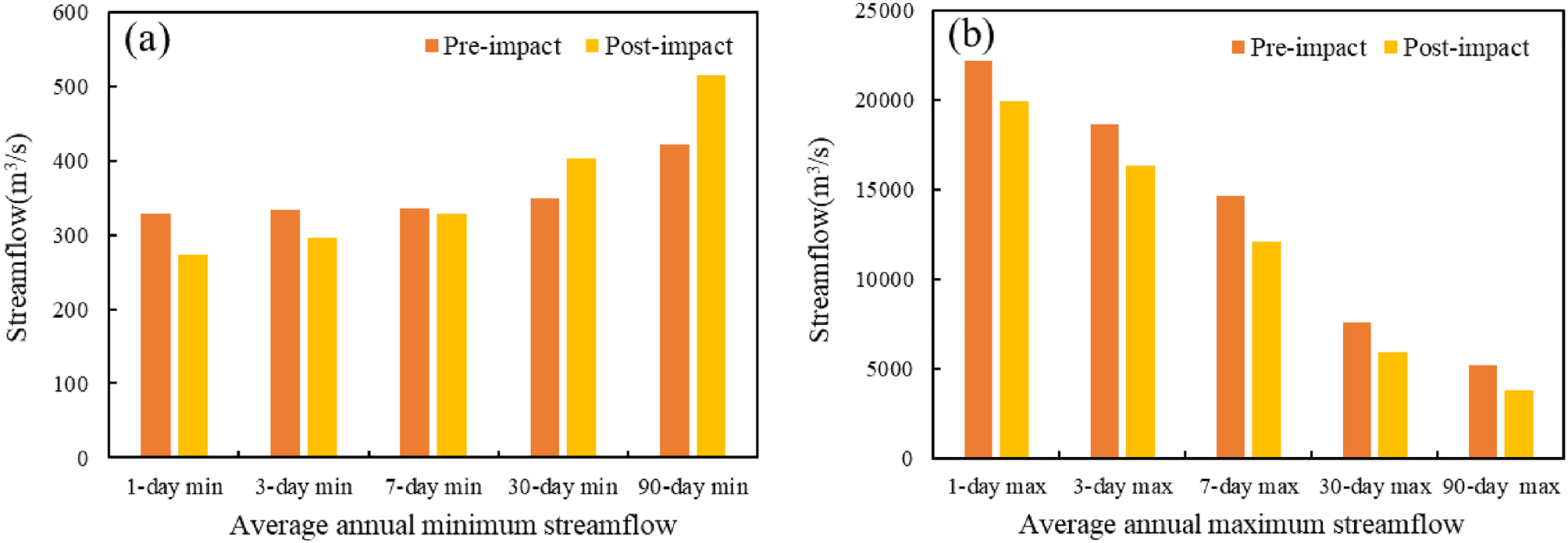
(**a**) Minimum streamflow alteration. (**b**) Maximum streamflow alteration.

1. Monthly median streamflow magnitude. Figure 3 shows the comparison of the monthly median streamflow before and after the alteration of the hydrological situation of the Jialing River. According to the comparison chart, it can be concluded that the changed Beibei station has different degrees of increase in streamflow from November to April and June of the coming year, among which the growth is more evident in January and March, and the degree of alteration reaches a high degree of alteration; while the monthly median streamflow from July to October and April has different degrees of decrease, and the degree of decline is more evident than the degree of increase. A high degree of alteration is reached in July, August, and September.
2. The magnitude of the extreme annual streamflow. Figure 4 shows the extreme yearly streamflow alteration. As can be seen from the figure: the minimum 1, 3, and 7d streamflow have decreased to different degrees, while the minimum 30d and 90d streamflow show an increase, in which the annual average minimum 1d streamflow has an apparent decreasing trend, and the degree of alteration reaches 66%, which is close to a high degree of alteration; the annual extreme values after the impact are all lower than those before the effect. This is consistent with the results of the M–K trend test that the runoff depth of Jialing River shows a decreasing trend.
3. The time of occurrence of extreme annual streamflow. The yearly minimum and maximum streamflow occurrence times at Beibei hydrological station are changed by less than 33%, which are mildly changed. Among them, the time of the maximum runoff is delayed by 11d, which indicates that the construction of the Jialing River Reservoir has made the high streamflow time within a stable range.
4. Frequency and duration of high and low streamflow. The number of high pulses and the calendar time of Beibei station both have different degrees of decrease, among which the number of high pulses decreased from 9 to 8, the degree of alteration was 24%. The calendar time of high pulses fell from 6 to 5d, the degree of alteration is 16%, both of which are less than 33%, which is a mild alteration; while the number of low pulses increased from 2 to 13, the degree of alteration is 82%, which is a high chance, and the calendar time of intense pulses decreased from 37 to 2d, with 63% alteration.
5. The streamflow rate of alteration and frequency. Beibei hydrological station runoff rate of rising hydrological alteration degree and fall rate hydrological alteration degree is 32% and 36%, respectively, both close to moderate alteration. The number of reversals has increased significantly compared with before the impact, and the degree of alteration has reached 100%. Since the ecosystem has limited ability to recover from external influences, the frequency alteration of Jialing River runoff will significantly impact the ecosystem in its basin^24^.

**Figure 5:**
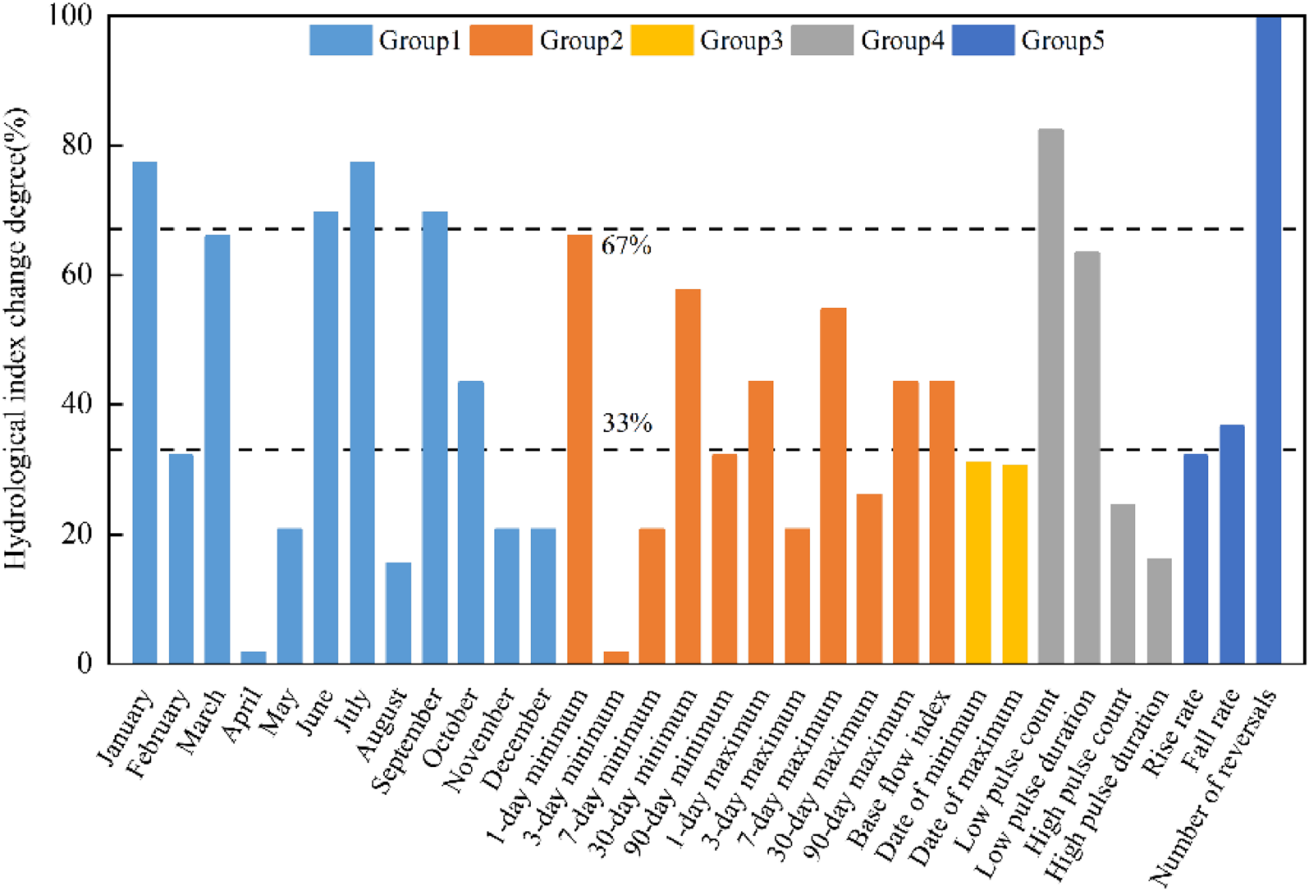
Hydrological alteration degree of Beibei station (Group 1 is the median monthly streamflow; Group 2 is the annual extreme streamflow; Group 3 is the time of occurrence of annual extreme value condition; Group 4 is the frequency and duration of high and low pulses; Group 5 is the rate and frequency of alteration in conditions).

Based on the IHA-RVA method for the analysis of the results before and after the mutation of the hydrological situation of Jialing River, the calculated results of 32 hydrological indicators before and after the mutation of Beibei hydrological station were absolute values and grouped with reference to Table 7, and the results are shown in Fig. 5. The hydrological indicators of moderate alteration reach 10, accounting for 32% of the total hydrological indicators, among which the median streamflow in March and the annual average minimum 1d streamflow hydrological alteration degree reaches 66%, close to the significant alteration; the hydrological indicators of low alteration reach 16, accounting for 50% of the total hydrological indicators. We calculate the overall hydrological index alteration degree and the hydrological index alteration degree of each group. The specific results are shown in Table 3. According to the results of the table can be concluded: Beibei hydrological station except for the third group of indicators for low alteration degree, the rest belong to medium alteration degree; the fifth group alteration degree is 64%, close to significant alteration; the overall hydrological index alteration degree is 50%, belong to medium alteration.

**Table 3:** Overall hydrological alteration of discharge sequence Unit (%).

Hydrological station	Hydrological alteration degree of each group					Overall hydrological alteration degree
	Group 1	Group 2	Group 3	Group 4	Group 5	
Beibei	51(M)	43(M)	31(L)	54(M)	64(M)	50(M)

### Attribution analysis of runoff alterations

The formulae based on the six Budyko assumptions calculate the contribution of different influencing factors, and the calculation results are shown in Table 4. According to the data in the table, the ranges of runoff depth alteration caused by rainfall, potential evapotranspiration, and human activities are − 32.96 to − 45.29 mm, 7.28–13.62 mm, and − 32.34 to − 38.28 mm, respectively, and the mean values are − 39.017 m, 10.288 mm, − 35.408 mm, so the mean value of runoff depth alteration calculated based on the six Budyko hypothesis formulas is − 64.13 mm. While the actual runoff depth alteration in the study area is − 64.01 mm, and the error range between them is 0.11 mm, indicating that the Budyko hypothesis-based hydrothermal coupling equilibrium principle of the elasticity coefficient method applies the runoff alteration attribution analysis in this study area. The contribution of precipitation to runoff alteration ranged from 51 to 71%, with a mean value of 61%; the gift of potential evapotranspiration to runoff alteration ranged from − 11 to − 21%, with a mean value of − 16%; and the contribution of human activities to runoff alteration ranged from 51 to 70%, with a mean value of 55%.

**Table 4:** Attribution analysis of hydrological regime.

Indicators	Zhang	Fu	Schreiber	Budyko	Pike	Ol’dekop	Average value
$\Delta R_{P} {\text{/mm}}$	− 37.30	− 38.91	− 38.03	− 32.96	− 41.59	− 45.29	− 39.01
$\Delta R_{E} /{\text{mm}}$	9.35	10.17	9.73	7.28	11.55	13.62	10.28
$\Delta R_{H} /{\text{mm}}$	− 36.10	− 35.58	− 35.86	− 38.28	− 34.26	− 32.34	− 35.40
$\eta_{P} /\%$	58	60	59	51	65	71	61
$\eta_{E} /\%$	− 14	− 15	− 15	− 11	− 18	− 21	− 16
$\eta_{H} /\%$	56	55	56	70	54	51	55

## Discussion

### Impact of climate alteration on runoff

Climate change and human activities are the two main driving forces affecting river flows^25^. Among them, climate change directly affects evapotranspiration, precipitation, and plant use efficiency, which in turn affects changes in hydrological regimes such as basin runoff and flooding. Xie et al. showed that the upper Yangtze River has experienced a significant increase in temperature at a rate of 0.16 °C per decade since 2003, resulting in greater evapotranspiration generation in the basin as well as a shorter duration and increased intensity of precipitation^26^. The IHA-RVA analysis shows that the discharge of the Jialing River varies greatly in magnitude, frequency, duration and flow velocity, which is mainly caused by the uneven distribution of precipitation and frequent rainstorms in the basin.

**Figure 6:**
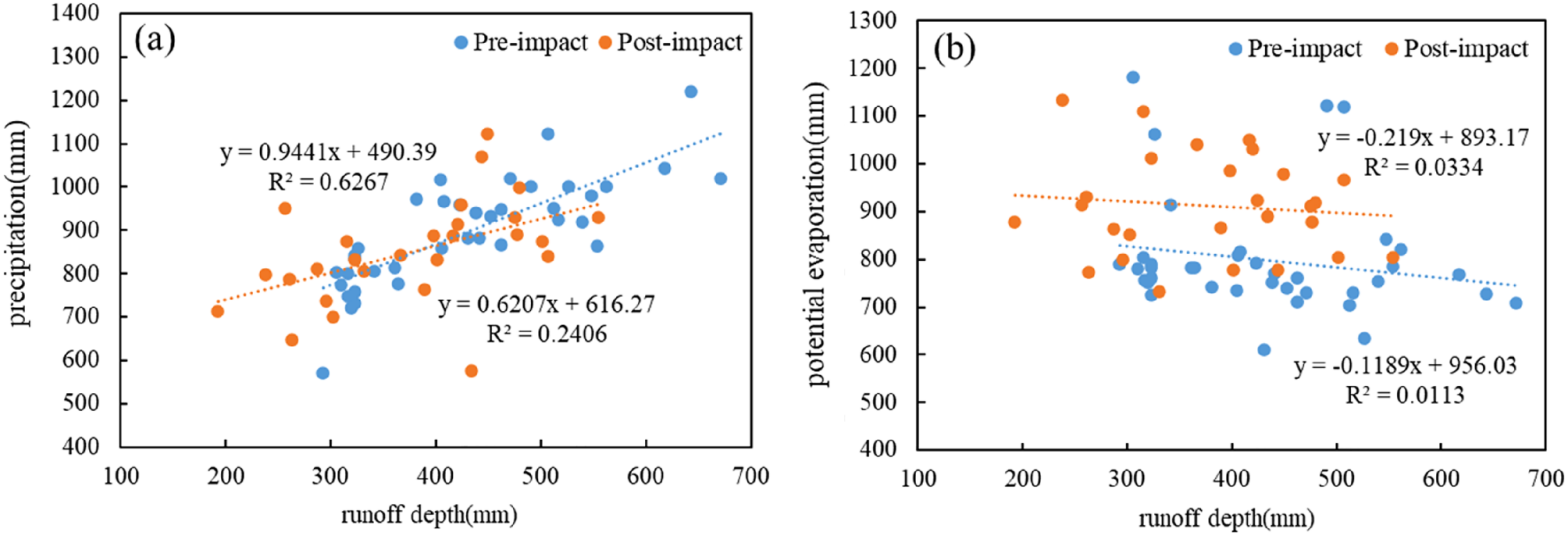
(**a**) Relationship between runoff depth and precipitation. (**b**) Relationship between Runoff Depth and Potential Evapotranspiration.

To further investigate the degree of influence of different climatic factors on the variation of runoff depth, the runoff depth was correlated with precipitation and potential evapotranspiration, respectively, and the results are shown in Fig. 6. As can be seen from Fig. 6a, the growth rate of precipitation with runoff depth before and after the mutation decreases from 0.944 to 0.621, and the correlation coefficient decreases from 0.627 to 0.241 from the base period to the change period, indicating that the change of Jialing River runoff is gradually weakened by the influence of precipitation. From Fig. 6b, it can be seen that the changes of potential evapotranspiration and runoff depth in the Jialing River basin from 1956 to 2020 have opposite trends, indicating that the runoff depth decreases with the increase of potential evapotranspiration; from the base period to the change period, the growth rate of potential evapotranspiration with runoff depth increases from 0.119 to 0.219 before and after the mutation, and the correlation coefficient increases from 0.0113 to 0.219. However, even though the correlation coefficient increased, the correlation coefficient between potential evapotranspiration and runoff depth was more different than that between precipitation and runoff depth, indicating that the change of Jialing River runoff was more influenced by rainfall.

Therefore, precipitation in the climate change of the Jialing River basin is the main influencing factor leading to runoff changes, which is consistent with the results of Guo et al. who predicted that runoff changes in the Yangtze River basin are mainly influenced by precipitation and that changes in potential evapotranspiration play a relatively small role in determining runoff changes^6^. However, Guo et al. showed that the influence of climate change on runoff is gradually weakening as external influences continue, and human activities are gradually dominating runoff changes^27^.

### Impact of human activities on runoff

The impact of human activities on runoff is mainly manifested by changes in the subsurface. Related studies have shown that the subsurface of a basin is mainly related to factors such as topography, soils, land use, vegetation and reservoirs^28^. At long time scales, factors such as soils and topography in the watershed are basically stable, and changes in the subsurface are mainly related to land use and vegetation cover changes and reservoir construction.

### Construction of reservoirs

It is well known that reservoir construction has a strong influence on river flows. Tang et al. showed that the regulation of reservoir construction on rivers is mainly reflected in the seasonal scale^29^. And IHA-RVA analysis shows that the monthly mean streamflow variation of Jialing River is high, especially from July to September, when the degree of alteration reaches a high degree of alteration (Table 2); the same variation is observed for all annual extreme streamflow, as well as an increase in extreme low flows and a decrease in extreme high flows (Fig. 4), where the annual minimum 1d flow reaches a high degree of alteration. The construction of the Jialing River reservoir (Table 5) on the one hand regulates the distribution of river flow during the year, making the flow more balanced in each month, cutting the flow during the flood season (June–October), while appropriately supplementing it during the non-flood season (January–May and November–December), and regulating the high and low river flows on an interannual basis, ensuring that the extreme river flows are within a stable range, which may be an important reason for the changes in the hydrological situation of the Jialing River. On the other hand, the construction of reservoirs increases the human water consumption (e.g. water supply, hydropower supply and irrigation), as well as the increase in evaporation due to the increased area of the water storage body and thus the loss of water, which further increases the water loss.

**Table 5:** Construction of main reservoirs in Jialing River Basin.

Reservoir	Position	Controlled drainage area (km^2^)	Operation time	Storage capacity (km^3^)
Shengzhong	Xi river	1756	1984	1.34
Baozhushi	Bailong river	28,428	1996	2.55
Caojie	Jialing river	156,100	2011	2.22
Tingzikou	Jialing river	62,550	2013	4.12

The river is a hydrologically connected ecosystem. With the completion of the construction of various types of reservoirs in the Jialing River basin, the connection between the main stem of the Jialing River and its major tributaries is blocked and the river flow is regulated^30^. Therefore, some small reservoirs with a large and inefficient area of influence were removed so that their river connections could be repaired and the natural flow regime could be restored to provide a reasonable habitat for some endemic and endangered fish species in the upper reaches of the Yangtze River such as migratory and adapted to rapid water flow^31^.

### Land use change

**Figure 7:**
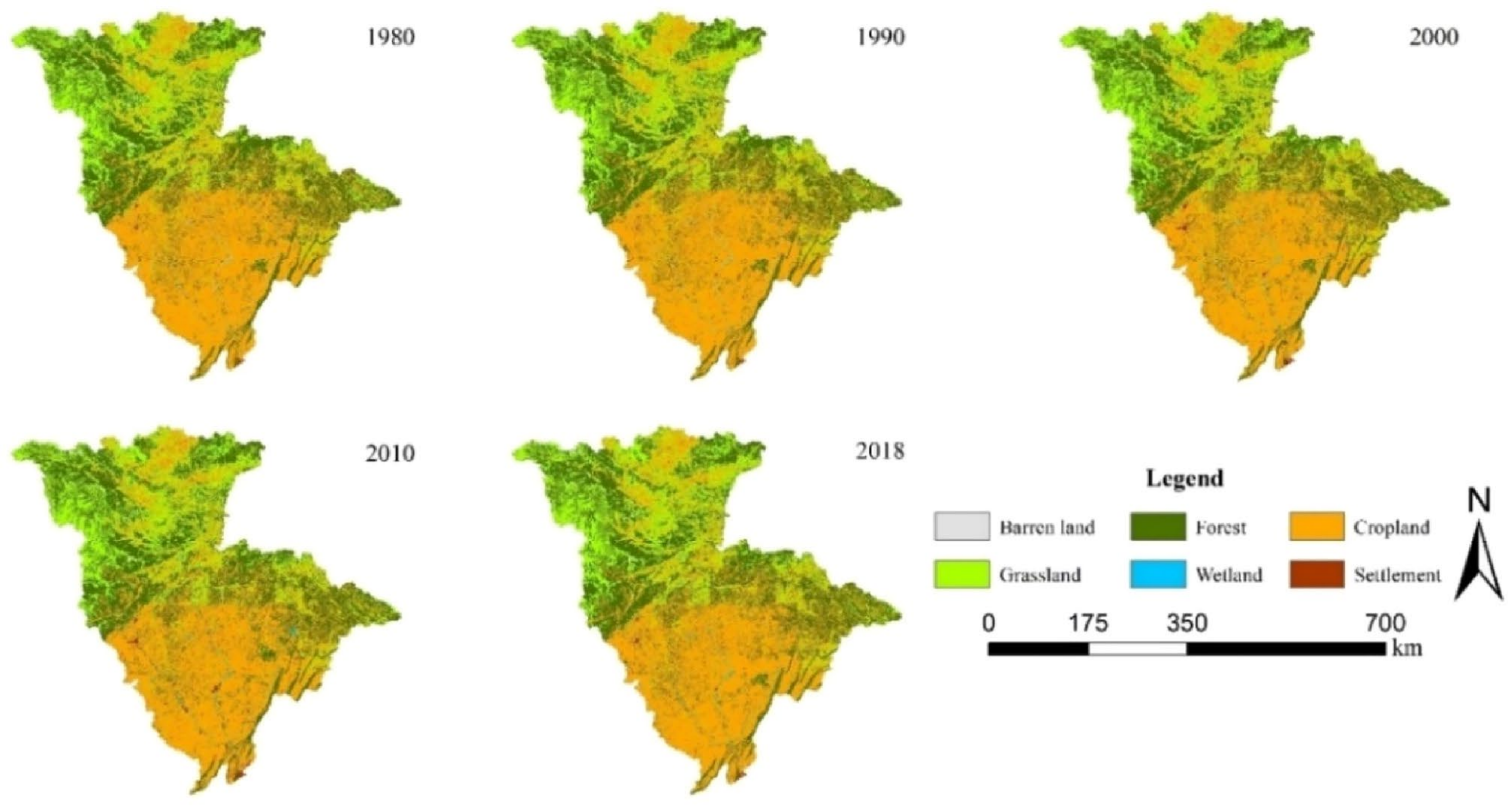
Land use change map of Jialing River Basin from 1980 to 2018 (The map was generated with data available from the China Resources and Environmental Science and Data Center using ESRI’s ArcGIS (version 10.1; https://www.resdc.cn/data.aspx)).

Chen et al. analyzed the effects of climate change, land use, and water conservancy projects on the Jialing River in the Yangtze River basin over the past 60 years, indicating that land use changes may cause environmental problems such as spatial and temporal changes in water resources, soil erosion, or low-end weather events in the basin^32^. Figure 7 and Table 6 show the distribution of land use in the Jialing River basin for different periods from 1980 to 2018. According to the information in the table, it can be seen that: grassland, cropland, and barren land in the basin have all decreased to different degrees, while forest, settlement, and wetland have all increased to different degrees; the conversion of cropland into the forest is the most prominent in the process of land use change, and its alteration area accounts for 7% of the whole basin area. The Jialing River runoff showed a decreasing trend after the mutation (after 1985) and a redistribution of monthly flow, indicating that hydrological processes change with land use changes. The conversion of a large amount of arable land to forest land in the Jialing River basin has caused the soil in the basin to have a deterrent effect on its runoff, which in turn leads to a decreasing trend in runoff.

**Table 6:** Land use conversion matrix of Jialing River from 1980 to 2018 (unit: km^2^).

1980\2018	Grassland	Settlement	Cropland	Forest	Wetland	Barren land	Total
Grassland	18,850.57	231.24	7635.86	8491.26	138.83	179.65	35,527.43
Settlement	141.66	199.40	396.87	85.60	29.93	–	853.48
Cropland	7283.87	1877.84	50,301.90	11,292.89	1020.73	23.58	71,800.84
Forest	7850.85	207.68	10,747.06	31,115.11	174.40	101.44	50,196.56
Wetland	115.54	106.49	748.26	141.63	292.08	2.94	1406.96
Barren land	215.77	3.02	59.11	170.59	6.03	174.76	629.30
Total	34,458.29	2625.68	69,889.09	51,297.09	1662.02	482.39	160,414.59

### Vegetation cover

**Figure 8:**
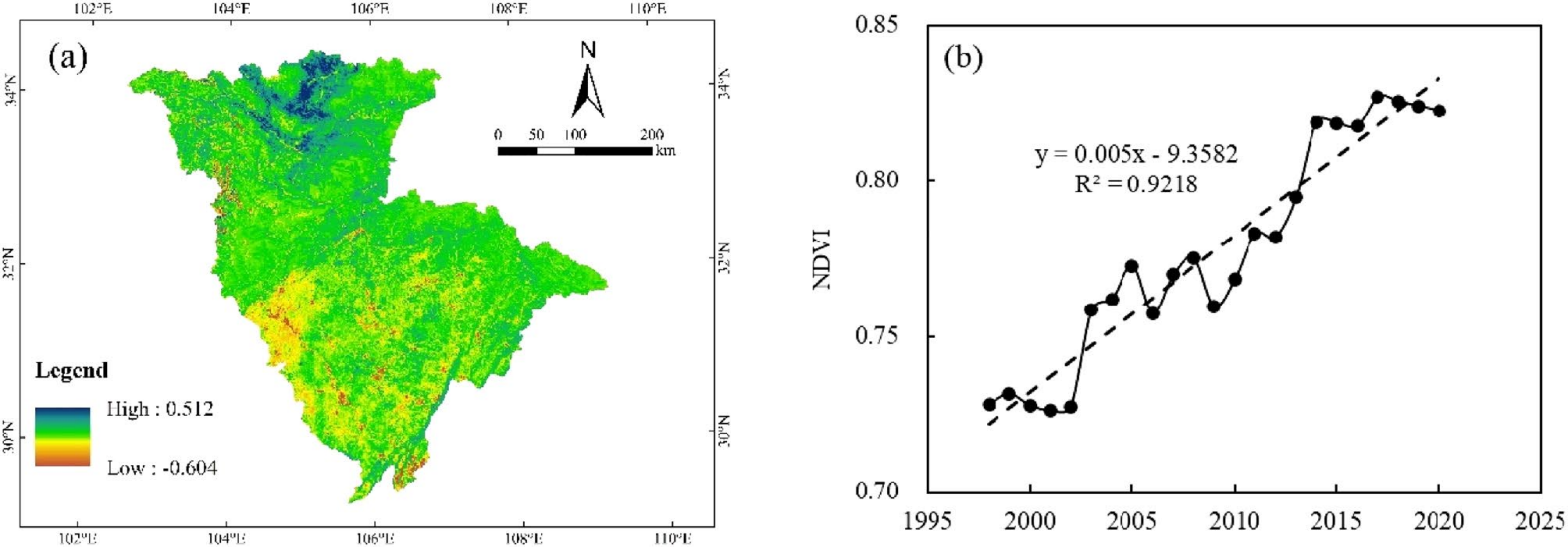
(**a**) Temporal and Spatial Distribution of Vegetation Coverage in Jialing River Basin. (**b**) Interannual alteration of NDVI in Jialing River Basin. (The map was generated with data available from the China Resources and Environmental Science and Data Center using ESRI’s ArcGIS (version 10.1; https://www.resdc.cn/data.aspx)).

To reveal the cover of the Jialing River basin, the normalized vegetation index (NDVI) was used to describe the vegetation cover alteration process. Figure 8 show the results of spatial and temporal alterations of NDVI in the Jialing River basin, from which it can be seen that: the alterations of vegetation cover in the basin are mainly concentrated in the northern area of Jialing River, while the southern site does not show large fluctuations due to the gentle topography and urban concentration. According to the trend of NDVI values from 1998 to 2020, it can be seen that the NDVI values in the Jialing River basin show an increasing trend, indicating a gradual increase in vegetation cover from the 1990s, which is consistent with the results of NDVI increase in the Yangtze River basin simulated by Cui et al.^33^. Zhou et al. showed that due to the large-scale soil and water conservation measures implemented in southwest China, the increase in vegetation cover in the Jialing River basin has significantly increased, while the effect of increased vegetation cover on rivers in the basin is mainly focused on sediment changes, while the effect of runoff changes is not obvious^22^. However, related studies have shown that the increase in vegetation cover causes a decrease in runoff, and land use changes in the Jialing River basin also show a decrease in arable land and an increase in forest land, indicating an increase in vegetation cover. Therefore, the increase of vegetation cover in the Jialing River basin does not only have an impact on sediment change, but is also important for runoff change.

## Conclusions

1. During the period 1956–2020, the runoff in the Jialing River basin showed an overall decreasing trend, the precipitation showed an overall decreasing trend, the potential evapotranspiration showed an overall increasing trend, and the mutation in runoff occurred in 1985.
2. Compared with before the mutation, the monthly median flow after the mutation decreased in July-November and May, and increased in December–April and June; the annual maximum flow showed a decreasing trend after the mutation, the annual minimum 30 and 90d flow increased, and the annual minimum 1, 3 and 7d flow decreased; the extreme flow ephemeris showed a decreasing trend, the number of high flows decreased and the occurrence time was delayed, the number of low flows increased and The alteration range is about 10d; the rising rate, fall rate and reversal all increase; in general, the alteration degree of Hydrologic regime in Jialing River basin is 50%, which is moderate alteration.
3. Precipitation is the dominant factor for runoff alteration, with its contribution to runoff alteration of 61%, and the contribution of human activities and potential evapotranspiration of 55% and − 16%, respectively. The influence of climatic factors on runoff alteration gradually decreases with time.
4. The obvious changes in the hydrological situation of the basin will lead to the damage to the ecological function of the Jialing River, which will have a greater impact on some fishes that are adapted to the habitat by drifting and fast currents.

## Study area and dataset

### Study area

Located between 29°–35°N and 102°–110°E, the Jialing River is one of the main tributaries of the upper reaches of the Yangtze River. The mainstream is 1120 km long, passing through Shaanxi Province, Gansu Province, and Sichuan Province, and finally joins the Yangtze River in Chongqing City, with a basin area of about 16 × 10^4^ km^2^, accounting for 15.9% of the Yangtze River basin area, and is a critical first-class tributary on the north bank of the Yangtze River, as well as the closest first-class tributary to the Three Gorges reservoir area in the upper reaches of the Yangtze River. The Beibei Hydrological Station is located in the lower reaches of the Jialing River. It is the export-controlling hydrological station of the Jialing River Basin, controlling 98% of the Jialing River Basin^34^. The hydrological data from Beibei hydrological station are usually used to analyze the changes of the hydrological situation of Jialing River^35^.

### Datasets

**Figure 9:**
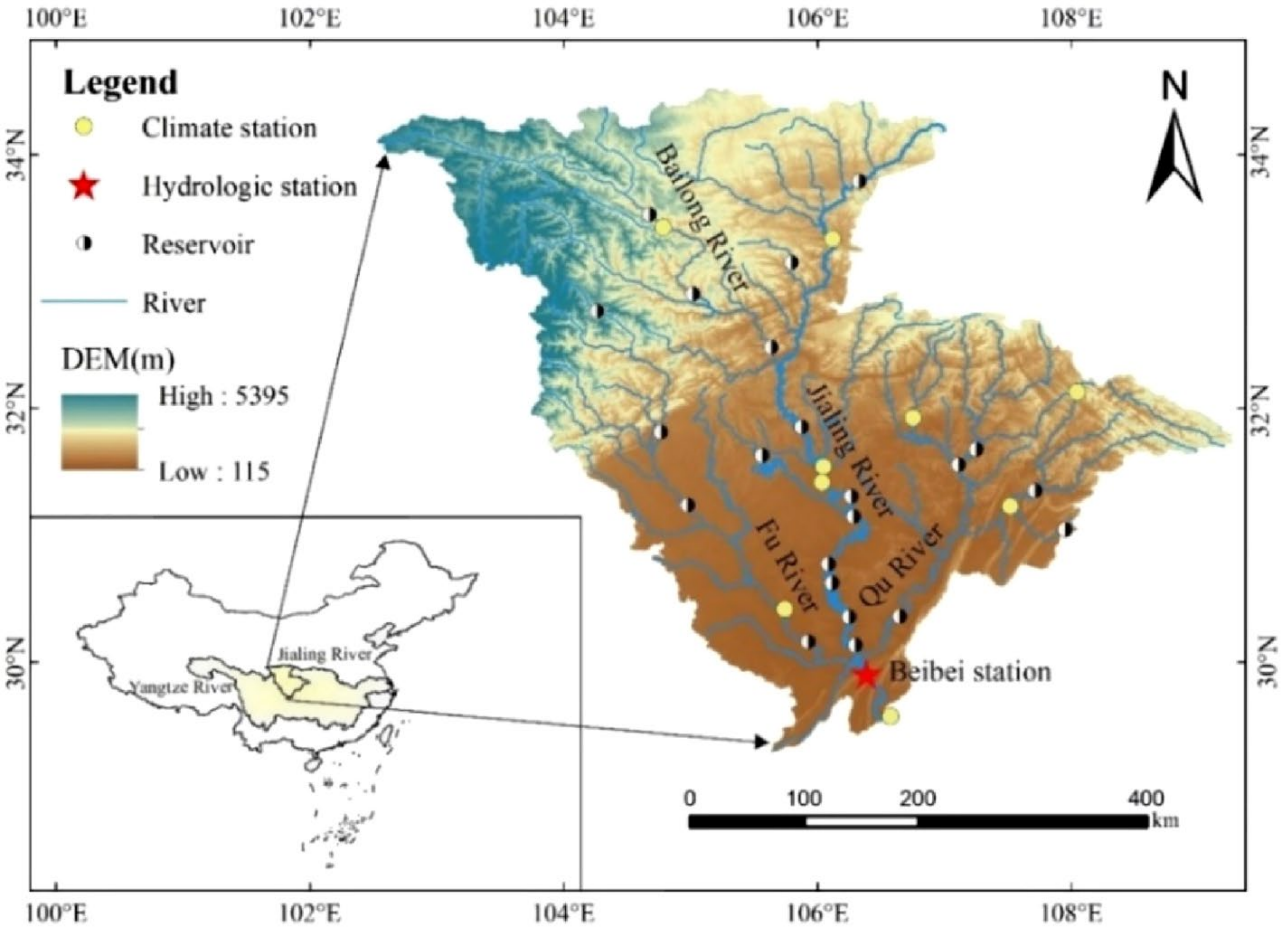
Jialing River Basin Map (the map was generated with data available from the Chinese Geospatial Data Cloud using ESRI’s ArcGIS (version 10.1; http://www.gscloud.cn/)).

The daily runoff data of the Jialing River-Beibei hydrological station from 1956 to 2020 used in this study were obtained from the "Hydrological Yearbook of Yangtze River Basin." For the meteorological data, meteorological data from nine meteorological stations (Fig. 9) in the Jialing River basin were used. The information was obtained from the China Meteorological Data Network (http://data.cma.cn/). The Normalized Difference Vegetation Index (NDVI) used in this paper is based on the gridded data from 1998 to 2020 at 1 km resolution. The monthly NDVI is first obtained according to the maximum method. Then the monthly NDVI is averaged to obtain the annual NDVI. The land use data are the national 1 km grid land use data for five periods of 1980, 1990, 2000, 2010, and 2018. The land use data and NDVI for each period were sourced from the Resource and Environment Science and Data Center of the Chinese Academy of Sciences (https://www.resdc.cn/data.aspx), and the data production was generated by manual visual interpretation using Landsat TM/ETM remote sensing images as the primary data source for each period.

## Methods

### Trend and mutation analysis

In this paper, the Mann–Kendall non-parametric test^36^ and the cumulative distance level method^37^ mainly analyze the trend and mutation of the daily streamflow data observed at the Beibei hydrological station. Among them, the Mann–Kendall nonparametric test is a more widely used method to test the abruptness of runoff. Still, its detection results have a significant human interference factor and need to be combined with other abruptness detection methods and the actual situation to ensure its accuracy. Please refer to the corresponding references for the calculation process^36, 37^.

### IHA-RVA method

To study rivers' complex hydrological alteration process, the hydrological alteration indicator method (IHA) proposed by Richter B.D and the range of alteration method (RVA) based on IHA are used in this paper to analyze the alteration process. Based on the day-by-day water level data of the Beibei hydrological station, 32 IHA hydrological indicators were calculated (since there was no daily streamflow of zero at the Beibei hydrological station during the study period, the hydrological indicator of zero discharge was not considered in this study). According to different ecosystem effects, the 32 hands were divided into five groups (Table 7). The hydrological indicators of streamflow, time, frequency, duration, and alteration rate were integrated to evaluate. The hydrological state of the river was changed. To quantitatively analyze the interference degree of different hydrological indexes, the following quantitative index formula is adopted.

1 $$D_{i} = \left| {\frac{{N_{oi} - N_{e} }}{{N_{e} }}} \right| \times 100\%$$

2 $$N_{e} = r \times N_{T}$$

where: $D_{i}$ is the hydrological degree of alteration of the $i$ IHA indicator, $N_{0i}$ and $N_{e}$ is the actual and desired number of years that fall within the RVA target threshold after the hydrological indicator alteration; $r$ is the proportion of the IHA falling within the RVA target threshold before the disturbance; $N_{T}$ is the total number of years after the hydrological indicator alteration.

**Table 7:** Indexes of IHA.

Group	IHA Parameters	Parameter index description
1	Median month	Median monthly streamflow
2	Annual pole size	Annual average 1, 3, 7, 30, 90d minimum and maximum streamflow (sand content), baseflow index^①^
3	Time of occurrence of annual extreme value condition	The date on which the maximum and minimum 1 day of the year occurs (Roman day)^②^
4	Frequency and duration of high and low pulses	Number of high and low pulses per year^③^ and average of pulse durations
5	Rate and frequency of alteration in conditions	Median annual values of increase (rate of increase) and decrease (rate of decrease) and number of reversals^④^

To set an objective criterion for the degree of alteration, it is stipulated that $D_{i}$ values between 0 and 33% are considered unaltered or low alteration; between 33 and 67% are considered a moderate alteration, and 67% to 100% are considered a high alteration. The overall degree of hydrologic alteration $D_{o}$ can be calculated using the following method.

3 $$D_{o} = \left( {\frac{1}{n}\sum\limits_{i = 1}^{n} {D_{i}^{2} } } \right)^{0.5}$$

where $n$ is the number of indicators, it is also specified that $D_{o}$ is between 0 and 33%, 33% and 67%, and 67% and 100% are low media, and high alterations, respectively^38^.① the ratio of the annual minimum continuous 7-day streamflow to the annual median; ② Roman day indicates the number of days in the calendar year; ③ low pulse is defined as the median of the day lower than 25% of the frequency before the disturbance, and high pulse is defined as the median of the day higher than 75% of the frequency before the disturbance; ④ the number of reversals refers to the number of times the daily streamflow turns from increasing to decreasing or decreasing to increasing.

### Estimation of potential evapotranspiration

In this study, the potential evapotranspiration is calculated using the FAO Penman–Monteith formula, which has been widely used by scholars:

4 $$ET_{0} = \frac{{0.408(R_{n} - G) + \gamma \frac{900}{{T + 273}}\mu_{2} \left( {e_{s} - e_{a} } \right)}}{{\Delta + \gamma \left( {1 + 0.34\mu_{2} } \right)}}$$

where: $ET_{0}$ is the potential evapotranspiration (mm/d); $\Delta$ is the slope of the saturation water pressure curve (Kpa/°C); $R_{n}$ is the net surface radiation (MJ/m^2^d); $G$ is the soil heat flux (MJ/m^2^d); $\gamma$ is the dry and wettable constant (Kpa/°C); $T$ is the average daily air temperature (°C); $\mu_{2}$ is the wind speed at 2 m (m/s); $e_{s}$ is the saturation water pressure (KPa); $e_{a}$ is the actual water pressure (KPa). The specific calculation process is described in reference^39^.

### Quantitative evaluation of runoff alterations

Runoff alterations in the basin should consider both climate and human activities^40, 41^ and are calculated as follows.

5 $$\Delta {\text{R}} = \Delta R_{P} + \Delta R_{E} + \Delta R_{H}$$

6 $$\eta_{{\text{P}}} { = }\Delta {\text{R}}_{P} /\Delta R \times 100\%$$

7 $$\eta_{{\text{E}}} { = }\Delta R_{E} /\Delta R \times 100\%$$

8 $$\eta_{H} { = }\Delta R_{H} /\Delta R \times 100\%$$

where: $\Delta {\text{R}}$ is the difference of runoff depth alteration; $\Delta {\text{R}}_{P}$, $\Delta {\text{R}}_{E}$, $\Delta R_{H}$ is the amount of runoff depth alteration caused by climate alteration and human activities; $\eta_{P}$, $\eta_{E}$, $\eta_{H}$ is the contribution rate of climate alteration and human activities.

The elasticity coefficient method^42, 43^ was chosen to calculate $\Delta R_{P}$, $\Delta R_{E}$ and $\Delta R_{H}$, based on the basin long time water balance equation (${\text{R = }}P - E_{\alpha }$), the dryness index ($\phi = E_{0} /P$) calculation and six formulas based on Budyko's assumptions (Table 8), as follows.

9 $$\Delta R_{P} = \varepsilon_{P} \frac{Q}{P}\Delta P$$

10 $$\Delta R_{E} = \varepsilon_{E} \frac{R}{{ET_{O} }}\Delta E$$

11 $$\varepsilon_{P} = 1 + \frac{{\phi F^{^{\prime}} \left( \phi \right)}}{1 - F\left( \phi \right)},\;\;{\text{and}}\;\;\varepsilon_{{E_{0} }} = 1 - \varepsilon_{P}$$

where: $Q$, $E_{0}$, $P$ and are the multi-year average runoff, potential evapotranspiration, and precipitation, respectively; $\Delta E_{0}$ and $\Delta P$ are the alterations of potential evapotranspiration and precipitation before and after the impact, respectively; $\varepsilon_{P}$ and $\varepsilon_{{E_{0} }}$ are the elasticity indices of runoff to precipitation and potential evapotranspiration, respectively.

**Table 8:** Six commonly used expressions based on Budyko hypothesis.

No	$F\left( \phi \right)$	$F^{^{\prime}} \left( \phi \right)$	Source
1	$F(\phi ) = \left( {1{ + }\omega \phi } \right)/\left( {1 + \omega \phi + 1/\phi } \right),\omega = 1$	$F(\phi )^{\prime} = \left( {\omega { + 2}\omega /\phi - 1 + 1/\phi^{2} } \right)/\left( {1 + \omega \phi + 1/\phi } \right)^{2}$	Zhang et al.^40^
2	$1 + \phi - \left( {1 + \phi^{a} } \right)^{1/a} ,\;a = 2.5$	$1 - \left( {1 + \phi^{a} } \right)^{1/a - 1} \phi^{a - 1}$	Fu et al.^41^
3	$1 - e^{ - \phi }$	$e^{ - \phi }$	Schreiber^44^
4	$\left[ {\phi \tanh \left( {1/\phi } \right)\left( {1 - e^{ - \phi } } \right)} \right]^{0.5}$	$$\begin{gathered} 0.5[\phi \tanh \left( {1/\phi } \right)\left( {1 - e^{ - \phi } } \right)]^{ - 0.5} \times [\tanh \left( {1/\phi } \right) - \hfill \\ {\text{sech}}^{2} \left( {1/\phi } \right)/\phi \left( {1 - e^{ - \phi } } \right) + \phi \tanh \left( {1/\phi } \right)e^{ - \phi } ] \hfill \\ \end{gathered}$$	Budyko^45^
5	$\left( {1 + \phi^{ - 2} } \right)^{ - 0.5}$	$1/\left[ {\phi^{3} \left( {1 + \left( {1/\phi } \right)^{2} } \right)^{1.5} } \right]$	Pike^46^
6	$\phi \tanh \left( {1/\phi } \right)$	$\tanh \left( {1/\phi } \right) - 4/\left[ {\phi \left( {e^{ - 1/\phi } + e^{1/\phi } } \right)^{2} } \right]$	Ol’dekop^47^
